# Ferroptosis-associated pathological injury mechanisms and therapeutic strategies after intracerebral hemorrhage

**DOI:** 10.3389/fneur.2025.1508718

**Published:** 2025-05-01

**Authors:** Yuhua Gong, Fumei Yang, Ying Liu, Yuping Gong

**Affiliations:** ^1^School of Smart Health, Chongqing Polytechnic University of Electronic Technology, Chongqing, China; ^2^Ultrasound Department of the Second Affiliated Hospital of Chongqing Medical University, Chongqing, China

**Keywords:** intracerebral hemorrhage, ferroptosis, iron overload, oxidative stress, inflammatory response

## Abstract

Intracerebral hemorrhage (ICH) is an important neurological disease caused by the rupture of blood vessels in the brain parenchyma, with a high mortality and disability rate. At present, many studies have focused on the injury mechanisms and intervention strategies after ICH. However, there is no effective clinical treatment that can significantly improve the prognosis of ICH patients. Ferroptosis, a regulated form of cell death, has been identified as a significant contributor to brain tissues damage and neurological dysfunction following ICH. The hallmark of ferroptosis is iron-dependent lipid peroxidation, which is closely related to the pathological process of iron overload and oxidative stress after ICH. Exploring the interaction between ferroptosis and pathological injury mechanisms post-ICH will contribute to our understanding the key pathways involved in the ferroptosis-related injury mechanisms and facilitating the discovery of appropriate intervention strategies. On this basis, we present a comprehensive overview of ferroptosis-related brain injury mechanisms (e.g., iron overload, oxidative stress, inflammatory response and mass effect) in the pathogenesis and development of ICH. Following ICH, the degradation of hematoma and iron metabolism provide the fundamental material basis for ferroptosis, and oxidative stress primarily participates in the lipid peroxidation process of ferroptosis via related molecular pathways (such as the GPX4). By synthesizing current evidence, this article aims to provide a theoretical foundation for future research on therapeutic strategies targeting ferroptosis and related pathways in ICH.

## 1 Introduction

Intracerebral hemorrhage (ICH) is a serious disease caused by the rupture of blood vessels in the brain parenchyma. This is a highly destructive subtype of cerebrovascular stroke that still faces significant challenges in clinical treatment. According to statistics, the 28-day mortality rate of ICH patients is as high as 47%, with a recurrence rate of approximately 25% among survivors in the next 5 years (1), and only 20% of ICH patients with independent living abilities after 6 months (2). The mortality and disability rates associated with ICH are considerably higher than those observed in other neurological emergencies. Although surgical removal of hematoma and control of elevated intracranial hypertension are biologically plausible interventions for ICH and life-saving measures in critical situations, improvements in mortality or functional outcome remain controversial (3). Therefore, it is of great practical significance to find effective intervention strategies to improve the clinical prognosis of ICH.

**Figure 1:**
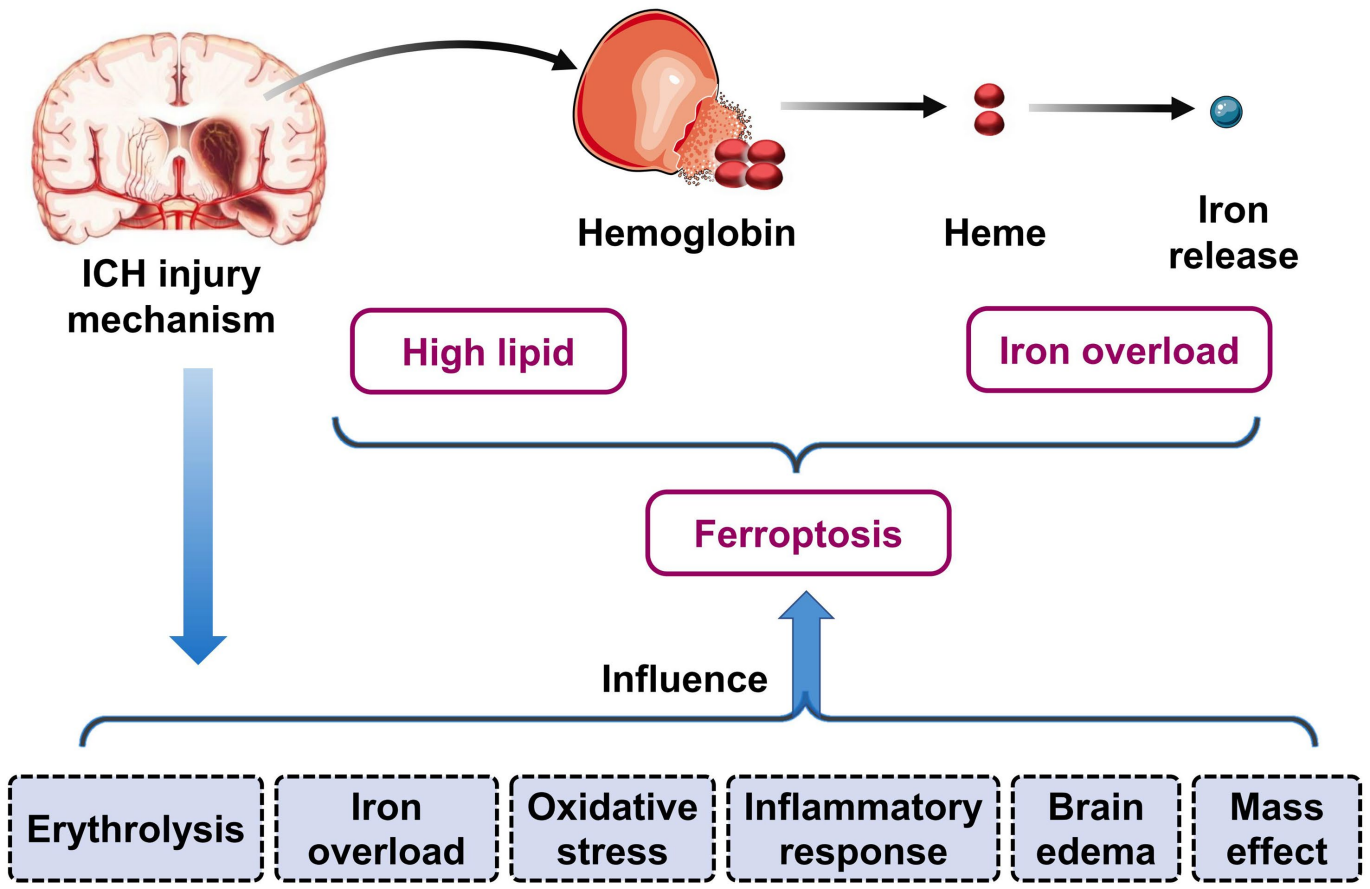
Injury mechanisms of intracerebral hemorrhage (ICH) associated with ferroptosis. Following ICH, the released free iron of degraded hematoma leads to the formation of a pathological microenvironment of iron overload in the perihematomal high lipid brain tissues. This constitutes the material basis for the occurrence of ferroptosis. In the process of ICH, multiple primary and secondary brain injury mechanisms (including erythrolysis, iron overload, oxidative stress, inflammatory response, brain edema, mass effect) can affect the process of ferroptosis.

The pathophysiological mechanism of ICH is complex and remains incompletely understood. Generally, the injury mechanism of ICH can be divided into two distinct phases: the primary brain injury and the secondary brain injury. The primary injury mainly refers to the mechanical compression and structural damage to brain tissues resulting from the rupture of blood vessels and the formation of hematoma, which is known as the mass effect. Mass effect will mechanically compress and stretch neurons and glia in the brain tissues immediately post-ICH, which will in turn lead to physical damage to the cellular structure (4, 5). Secondary brain injury is caused by the invasion of exogenous blood into the brain parenchyma, which gives rise to a series of mechanisms that result in injury to brain tissues (Figure 1). These include the release of toxic products from the erythrolysis, such as hemoglobin, heme, and free iron; oxidative stress; the physiological response of brain tissues to the toxic microenvironment in peri-hematoma (i.e., inflammatory processes); the coagulation cascade reaction; blood–brain barrier damage; and cerebral edema (6, 7). In the process of ICH, a multitude of primary and secondary injury mechanisms persist and interact with each other, ultimately resulting in neurological dysfunction and a poor prognosis for ICH patients.

Among the intricate mechamisms of ICH injury, iron-dependent ferroptosis, a regulated cell death form, plays a central role in brain tissues damage and treatment of ICH. With the development of ICH, the hemoglobin released by erythrolysis produces a large amount of free iron, which catalyzes the generation of reactive oxygen species (ROS) via the Fenton reaction, attacking lipids, proteins and DNA, eventually leading to irreversible neuronal damage and worsening neurological dysfunction. Ferroptosis represents an important mechanism of cell death following ICH, characterized by iron-dependent lipid peroxidation (8). Meanwhile, ferroptosis as the non-apoptotic form of cell death has been demonstrated to be associated with the pathogenesis of various diseases (9). After ICH occurrence, multiple ICH injury mechanisms (e.g., hematoma degradation, iron metabolism, oxidative stress, and inflammatory) can mediate ferroptosis in cells through different actions. Further exploration of the regulatory mechanism of ferroptosis after ICH will not only help to elucidate the molecular basis of brain injury, but also provide a theoretical basis for the development of sequential and multi-target intervention strategies. Therefore, this review provides a summary of the injury mechanisms related to ferroptosis after ICH and the latest intervention strategies (Table 1), with the aim of establishing a theoretical framework for the research and treatment of ICH.

**Table 1:** The reagents and mechanisms of action associated with ferroptosis.

Reagents/molecules	Target	Mechanisms	References
Deferoxamine	Iron	Reduced iron overload	(24)
Ferrostatin-1	Iron	Decrease of labile iron, disappearance of lipid hydroperoxides	(39)
Nedd4 overexpression	DMT1	Decreased DMT1 expression, reduced iron overload	(32)
Hepcidin	Iron transport proteins	Suppressed the increase of iron and ferritin	(35)
Interleukin-10	IL-10R/STAT3	Reprogrammed lipid metabolism, reduced ROS accumulation	(41)
Pioglitazone	PPARγ	Promoted GPX4 expression	(46)
Crocin	Nrf2	Reduced MDA content, increased GPX4 expression	(48)
Withaferin A	Nrf2/HO-1	Decreased the level of MDA and 4-HNE	(49)
Salvianolic acid A	Akt/GSK-3β/Nrf2	Increased Nrf2, GPX4 expression	(50)
Selenium	Nrf2	Increased GPX4 expression	(51)
FoxO3a knockout	HO-1	Alleviate microglial activation and ferroptosis	(27)
HDA1/2 inhibitor	Nrf2/HO-1	Promote microglial anti-inflammatory phenotype, reduced iron deposition	(54)

## 2 Mechanisms of ferroptosis

**Figure 2:**
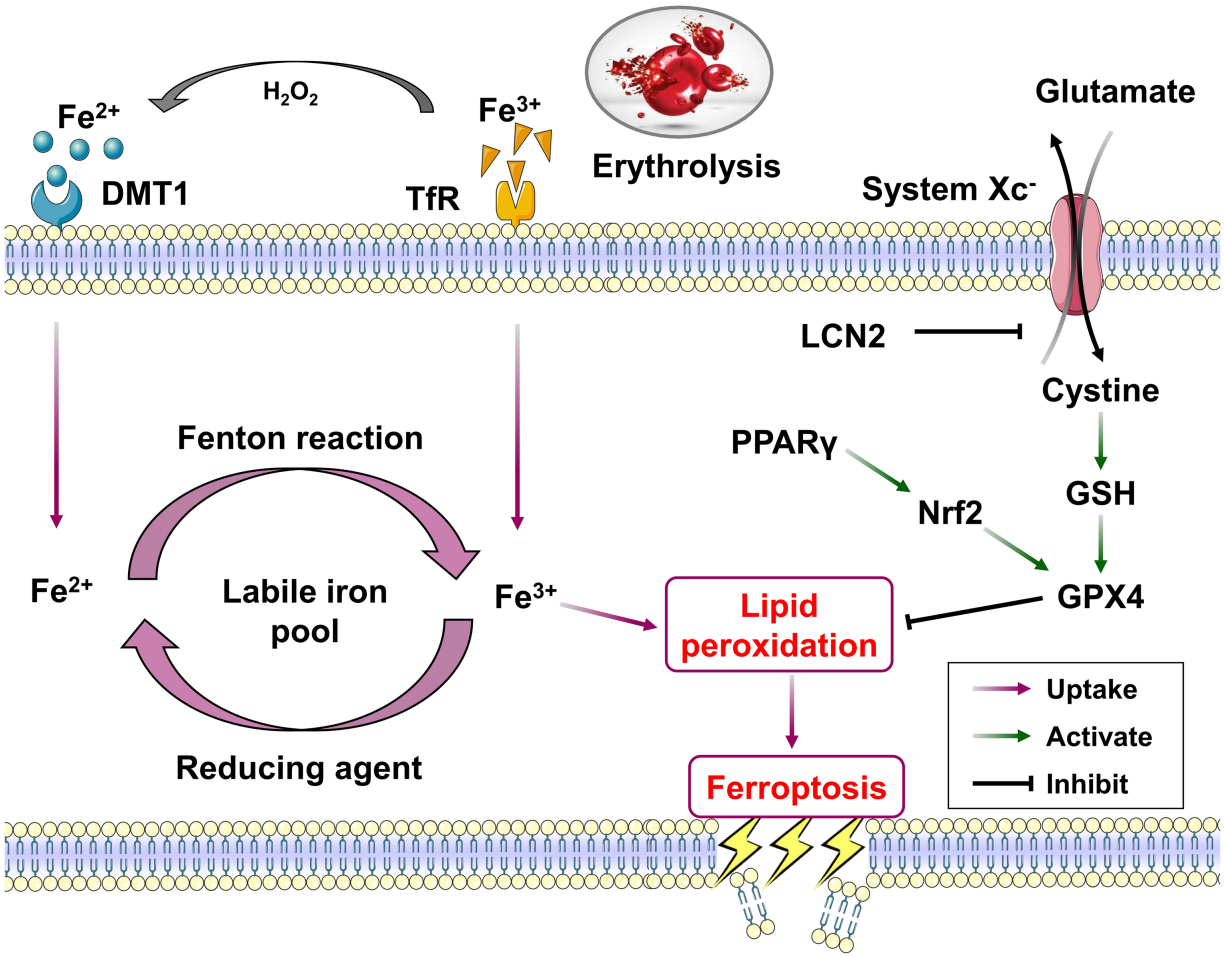
The core mechanisms and related pathways of ferroptosis post-ICH. Overloaded iron in peri-hematoma enters the cells via the uptake of TfR and DMT1, resulting in the form of labile iron pool to serve as the catalyst for the initiation of ferroptosis. Divalent iron (Fe^2+^) could generate highly toxic hydroxyl radicals through the Fenton reaction and induce lipid peroxidation. The system Xc^−^ and its downstream GSH/GPX4 pathway together constitute a critical reduction system against the lipid peroxidation of ferroptosis process. The activation of PPARγ/Nrf2 pathway could inhibit ferroptosis post-ICH via promoting GPX4 expression.

Ferroptosis is an important form of cell death after ICH. Distinct from the mechanism and morphology of apoptosis, necrosis and autophagy, ferroptosis is a unique regulated cell death modality, that is characterized by iron-dependent lipid peroxidation, and mainly driven by iron accumulation, lipid peroxidation and subsequent plasma membrane disruption (Figure 2). Ferroptosis has obvious cell micromorphological changes that are distinct from other forms of cell death. The main features are mitochondrial shrinkage and increased membrane density, as well as the reduction or disappearance of mitochondrial cristae (10). In the process of iron-dependent lipid peroxidation, the free radical derived from the Fenton reaction of free iron can further react with polyunsaturated fatty acids (PUFAs) and result in the formation of phospholipid hydroperoxides (PLOOH) on cell membranes. PLOOH is the product and main initiator of this oxidation reaction, and is also an activator of lipoxygenase. Glutathione peroxidase (GPX4) can prevent ferroptosis by directly neutralizing PLOOH through a glutathione (GSH) dependent reduction reaction and is the hallmark protein and important regulator of ferroptosis (11, 12). When the expression of GXP4 is dysregulated or relatively insufficient, it will lead to cellular ferroptosis through the accumulation of ROS (13). Therefore, GSH-GPX is considered as the main pathway for anti-ferroptosis.

## 3 Triggering effect of ICH pathological changes on ferroptosis

The pathophysiological changes post-ICH can be categorized into two key stages: primary brain injury and secondary brain injury. The primary brain injury is mainly caused by the mass effect formed by hematoma, resulting in local cerebral ischemia, hypoxia and physical destruction. Mass effect can cause the rapid erythrolysis and the release of substantial amounts of hemoglobin, which can be converted to free iron via the Heme oxygenase-1 (HO-1) metabolic pathway, thus providing the material basis for the occurrence of ferroptosis (14). Concurrently, in the acute phase after ICH, direct hematoma compression can cause the collapse of the mitochondrial membrane potential and elicit the hallmark features of ferroptosis, including the disappearance of mitochondrial cristae and membrane lipid peroxidation. It is important to note that the mitochondrial dysfunction induced by primary brain injury also promotes the transformation from their primary role of adenosine triphosphate (ATP) production to the predominant ROS generation. More than that, ATP depletion can lead to the obstruction of GPX4 synthesis, which further weakens the scavenging activity of nerve cells to ROS (15).

Secondary brain injury typically develops gradually over the course of hours to days following ICH. The core mechanism of secondary damage involves a cascade of oxidative stress initiated by iron overload. With the continuous degradation of hemoglobin, free iron produces a significant quantity of ROS through Fenton reaction. These ROS can attack PUFAs in cell membranes and form PLOOH, and result in ferroptosis (16). Meanwhile, the activation of inflammatory response post-ICH also can further aggravate the ferroptosis process. Pro-inflammatory factors released by activated microglia, such as TNF-*α* and IL-6, can inhibit the function of the cystine/glutamate antiporter (system Xc^−^) and lead to inadequate GSH synthesis and GPX4 inactivity, thereby mediating ferroptosis (17). Generally, both the primary and secondary brain injury pathological process have regulatory effects on ferroptosis post-ICH. Primary brain injury tends to affect the material basis of ferroptosis (such as iron ions and mitochondria) through direct mechanical stimulation. For the secondary brain injury, multiple mechanisms (i.e., oxidative stress, inflammatory response) are more likely to involve in the chemical reaction and molecular regulation process of ferroptosis through different action pathways.

## 4 Injury mechanisms associated with ferroptosis after ICH

### 4.1 Hematoma and its degradation products

In the process of secondary brain injury after ICH, the hemoglobin and heme derived from erythrolysis can release free iron into the surrounding brain tissues via a variety of metabolic pathways, which together create a pathological microenvironment of iron overload around the hematoma. Iron overload is recognized as an important mechanism of injury in the secondary brain injury. Its accumulation in the peri-hematomal area can lead to the production of lethal ROS and the formation of lipid peroxidation, and induce a variety of other secondary brain injuries, including mitochondrial damage, brain edema and cell death (18, 19). Free iron ions are present in brain tissues in both divalent and trivalent forms. Divalent iron can generate highly toxic hydroxyl radicals through the Fenton reaction. These radicals are responsible for the mitochondrial damage, lipid membrane dysfunction, cell death, as well as neurological dysfunction (8). The trivalent iron that is produced as a result of the Fenton reaction will be reduced to divalent iron by the action of reducing agents, thereby forming a cyclical process of free radical generation (Figure 2) (20). It is therefore considered that iron overload, formed by the hematoma and its degradation products, is an important factor for the process of lipid peroxidation in brain tissues, the production of lethal ROS and ferroptosis of nerve cells (8, 21). The reduction of iron overload represents an efficacious treatment strategy for ICH. A number of preclinical studies have demonstrated that a range of iron chelators, including deferoxamine, minocycline and deferiprone, can effectively mitigate the damage caused by iron overload in the context of ICH (22–24). Deferoxamine treatment has been demonstrated to significantly improve neuronal damage, brain atrophy, brain edema, DNA damage, microvascular spasm and promote neurological function recovery after ICH (25, 26). The production of free iron in the peri-hematoma is directly correlated with the hematoma volume and the degradation and metabolism of hematoma. It has been shown that FoxO3a is highly co-localized with neurons and microglia in mice following ICH. Heme released from hematoma has been verified to enhance microglial polarization to the M1 proinflammatory phenotype and aggravate ferroptosis by stimulating the expression of FoxO3a/HO-1 pathway in microglia (27). Furthermore, iron deposition and ferroptosis are reported to prevalent in the long-term progression of ICH. The administration of ferrostatin-1, a ferroptosis inhibitor, has been shown to markedly reduce neuronal death in organotypic hippocampal slice co-cultured with hemoglobin, heme, or divalent iron. The inhibition of ferroptosis has been observed to considerably enhance the prognosis of ICH in a collagenase injected mouse model (18, 28).

### 4.2 Iron transport and metabolism

The substantial quantity of free iron accumulated around the injured cerebral tissues following ICH can enter the cells via the uptake of transferrin receptor (TfR) and divalent metal transporter 1 (DMT1). The formation of an excess of iron ions within the cell results in the labile iron pool, which serves as the catalyst for the initiation of ferroptosis (9). It has been demonstrated that neurons are more vulnerable to iron overload damage compared with macrophages/microglia. This is primarily due to the fact that neurons lack an adequate iron chelation system to cope with the overloaded iron, which results in the intracellular iron ions not being sequestered effectively, and then continuously produces the hydroxyl radicals through Fenton reaction (29). Additionally, the higher expression of iron uptake-related proteins (i.e., TfR and DMT1) in neuronal cells compared to glial cells (astrocytes, microglia and oligodendrocytes) is also considered to be a key factor contributing to their susceptibility to iron overload (30). Xie et al. reported that iron chelators can reduce the occurrence of ferroptosis in nerve cells by reducing the levels of iron overload mediated by the TF/TfR pathway (31). A reduction in DMT1 expression in brain tissues was observed following the overexpression of neuronal precursor cells expressed developmentally down-regulated 4 (Nedd4), which significantly inhibited the ferroptosis post-ICH (32). Iron overload represents a significant contributing factor to the induction of ferroptosis and other forms of secondary brain damage after ICH. Therefore, further research into the mechanisms of iron uptake and the potential intervention is of great significance for reducing secondary brain injury and promoting neurological function recovery following ICH.

Hepcidin, a key hormone secreted by the liver, plays a pivotal role in regulating iron homeostasis in the body. Hepcidin is capable of binding to the ferroportin (Fpn) of iron export protein, thereby regulating the output of dietary iron absorbed by enterocytes. This is achieved by regulating the internalization and degradation of the Hepcidin/Fpn complex, which in turn maintains iron homeostasis in the blood (33). Recent studies have revealed that hepcidin is also extensively distributed in brain tissues. Which can reduce iron overload in brain tissues by down-regulating iron uptake-related proteins (TfR, DMT1) and iron export protein (Fpn) in endothelial cells, neurons and glial cells to decrease iron transport from the periphery to brain and increase the export of iron from brain. Meanwhile, hepcidin intervention has been proved to markedly attenuate the elevation of iron in brain tissues subsequent to ICH through the inhibition of ferroportin expression. These has been shown to improve neural apoptosis, ROS production and neurological dysfunction following ICH (34, 35).

### 4.3 Oxidative stress

The presence of free iron has been found to facilitate the production of ROS and consequently lead to lipid peroxidation through the Fenton reaction. Concurrently, the oxidation of PUFAs also can generates lipid peroxides, which represent the fundamental process of ferroptosis (36). Subsequently, lipid peroxides will undergo further reactions with a number of reducing biomolecules, including proteins, lipids, and DNA. This will result in the enzymes inactivation, proteins destruction, DNA and phospholipid structures damage (37). Oxidative stress plays a pivotal role in the regulation of ferroptosis. The impairment of antioxidant systems can result in or accelerate lethal lipid peroxidation. This process can be effectively inhibited by various synthetic antioxidants, including ferrostatin-1 and liproxstatin-1 (38, 39). A considerable body of researches have documented the intervention strategies of regulating ferroptosis based on the inhibition of oxidative stress processes. Dexpramipexole has been certified to inhibit ferroptosis in peri-hematomal white matter by reducing the accumulation of iron and ROS (40). Another study has reported that Interleukin-10 (IL-10) can reduce the ferroptosis of oligodendrocyte progenitor cells by reprogramming lipid metabolism and reducing ROS accumulation (41). Furthermore, pharmacological agents such as baicalin, edaravone, and omarigliptin have been confirmed to inhibit brain ferroptosis and neurological impairment following ICH via the oxidative stress pathways (42–44).

Glutathione peroxidase (GPX4), as the key regulator and marker protein of ferroptosis, plays an important role in the process of brain injury and repairment following ICH. Studies have shown that GPX4 expression declines gradually post-ICH, reaches the lowest point at 24 h, and then exhibits a gradual increasement (45). This suggests that GPX4 is intimately associated with the progression of ICH. The peroxisome proliferator-activated receptor *γ* (PPARγ) agonist pioglitazone has been testified to promote GPX4 expression and inhibit neuronal ferroptosis and nerve injury following ICH by regulating the interaction between the PPARγ and Nuclear factor erythroid 2-related factor 2 (Nrf2) pathways (46). Additionally, malondialdehyde (MDA) and 4-hydroxynonenal (4-HNE), the secondary products of lipid peroxidation, are commonly used as reliable markers to evaluate lipid peroxidation, and are also employed as crucial indicators of ferroptosis (28, 47). Crocin intervention has been revealed to significantly reduced the MDA content and reversed GPX4 expression and neuronal ferroptosis following ICH (48). Zhou et al. have observed that Withaferin A can markedly suppress the heme-induced elevation in ROS and 4-HNE levels, as well as ferroptosis. Through the Nrf2 silence with siRNA or the use of a HO-1 inhibitor, these effects have been confirmed to closely related the Withaferin A activated Nrf2/HO-1 signaling pathway (49). Molecular docking and functional enrichment analysis, as well as an *in vivo* study, have demonstrated that Salvianolic acid A can effectively improve neuronal ferroptosis via enhancing the Akt/GSK-3β/Nrf2 signaling pathway activation (50). Bovine serum albumin-stabilized selenium nanoparticles (BSA-SeNPs) have been evidenced to effectively activate Nrf2 and enhance the expression of the antioxidant GPX4 based on the physiological functions of selenium, including scavenging free radicals and preventing oxidation, thereby inhibiting ferroptosis (51).

### 4.4 Inflammatory response

Microglia represent the primary endogenous phagocytic cells within the brain tissues, and they play a crucial role in iron metabolism, ferroptosis, and other secondary injury mechanisms following ICH. When ICH occurs, microglia are rapidly recruited to the periphery of the hematoma, subsequently undergoing a phenotypic transition from M1 to M2 (52). In contrast to the M1 phenotype, which exhibits high pro-inflammatory cytokine release and ferroptosis characteristics, M2 phenotype microglial cells demonstrate notable resistance to ferroptosis and neuroprotective properties (21). In addition to its inhibitory effect on ferroptosis, ferrostatin-1 has been proved to accelerate the M2 polarization of microglia, enhance their phagocytic function, and suppress the inflammatory response in mice after ICH (53). HO-1 is a key regulator of hemoglobin cleavage to heme, which contributes to the accumulation of neurotoxic iron. Studies have demonstrated that FoxO3a, which has a binding site with HO-1, plays a pivotal role in the regulation of inflammation and ferroptosis in ICH mouse model. Conditional knockout of FoxO3a expression in microglia can significantly alleviate microglial activation and ferroptosis induced striatal injury in mice (27). Regulation of Nrf2/HO-1 signaling pathway by the HDA1/2 inhibitor also has been demonstrated to significantly promote the anti-inflammatory phenotype of microglia and reduce iron deposition and ferroptosis after ICH (54). Targeting microglial S100A8 has an obviously beneficial impact on microglial autophagic-dependent ferroptosis after subarachnoid hemorrhage (55). Following ICH, activated microglia facilitate the infiltration of peripheral neutrophils and other immune cells into the periphery of the hematoma via chemokines, and neutrophils can secrete an important iron-binding glycoprotein, lactoferrin. It has been demonstrated that recombinant lactoferrin can mitigate neuronal ferroptosis by binding to ferric ions and reducing the intracellular iron concentration in autologous blood injected hyperglycaemic mice (56).

### 4.5 Other mechanisms

The primary mass effect post-ICH occurs immediately after the rupture of blood vessels and blood influx into brain tissues, and then it is accompanied by other secondary brain injury for a long time. A variety of injury mechanisms interact with one another to collectively influence the recovery process of the neurological function. In the early stages of ICH, mass effect can result in the rapid deposition of iron in brain tissues by accelerating erythrolysis of hematoma and releasing toxic hemoglobin (57). Subsequently, mass effect results in a notable upregulation of the mechanosensitive ion channels Piezo-2, pERK1/2 and iron uptake proteins (DMT1 and TfR). The mass effect mediated intracellular iron deposition and ferroptosis can be ameliorated by inhibiting the pERK1/2 pathway and reducing the expression of iron uptake proteins (DMT1 and TfR) (14). In addition, brain edema also exerts an inductive effect on ferroptosis. Studies have found that the rat ICH model with brain edema exhibits significant characteristics of lipid peroxidation and markedly regulates the expression of ferroptosis specific genes such as ACSL4 and SLC7A11 (58).

## 5 Interaction of ferroptosis with other cell death forms

In recent years, ferroptosis has attracted much attention as a regulated type of cell death. However, there are many other forms of cell death that have been identified and cannot be ignored in ICH process, including apoptosis, necrosis and autophagy (59). Apoptosis is characterized by cell shrinkage, chromatin condensation, and caspase activation, while necrosis is distinguished by cellular swelling and plasma membrane rupture. With regard to autophagy, it is hypothesized that there may be differential effects at varying stages of ICH. For instance, neuronal autophagy may be induced by the overactivation of autophagy. Conversely, autophagy may have a neuroprotective effect in later stages by way of the clearance of cellular debris (60). Actually, these forms of cell death may occur together post-ICH and collectively contribute to cells death and brain damage. Sometimes, the shrunken and swollen mitochondria can even coexist in the same neuronal soma (28). However, when cultured neurons are exposed to hemoglobin or hemin, the neuronal characteristics manifest as ferroptotic and necroptotic cell death, but not autophagy or caspase-dependent apoptosis (61). At present, the molecular mechanisms of how a cell selectively triggers different death processes under the same stress conditions have not been fully elucidated, and the cascades of regulatory networks and their spatio-temporal interaction patterns among these death pathways still need to be further explored. It is particularly noteworthy that p53, as an important regulatory node, can not only induce the expression of apoptosis-related genes, but also promote ferroptosis by inhibiting SLC7A11, reflecting the cross-regulation of different death pathways at the molecular level (62). In addition, autophagy has been demonstrated to lead to iron-dependent ferroptosis through degradation of ferritin and induction of TfR1 expression (63). Therefore, revealing the dynamic transformation rules of different cell death forms is of great practical significance for exploring cooperative intervention strategies and improving neuronal death after ICH.

## 6 The clinical application prospect of targeting ferroptosis post-ICH

**Figure 3:**
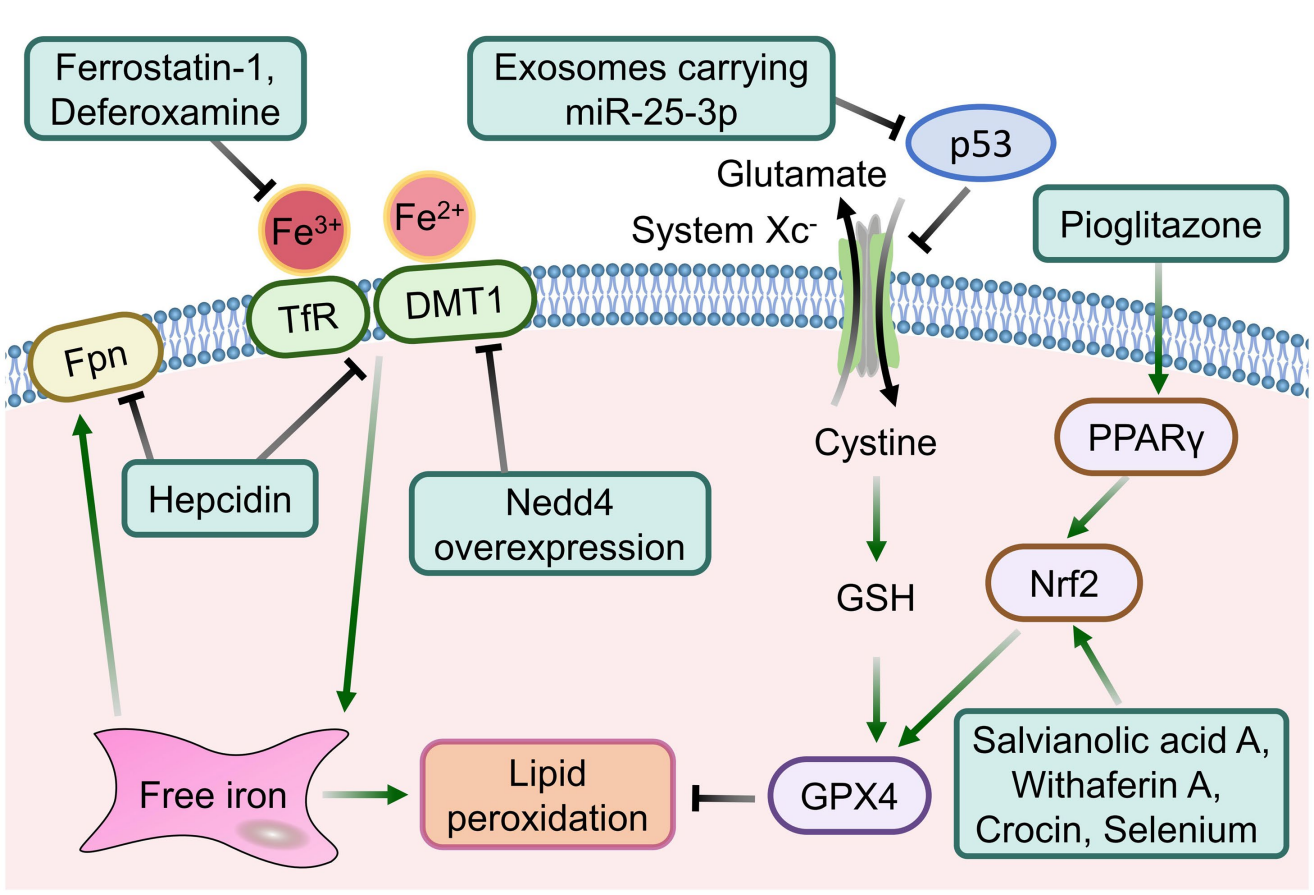
The intervention strategies targeting signaling pathways associated with ferroptosis following ICH.

Ferroptosis represents a form of regulated cell death. The development of intervention strategies targeting ferroptosis provide promising new avenues for the treatment of a range of diseases, especially central nervous system diseases that are highly susceptible to iron homeostasis imbalance, including Parkinson’s disease (64), Alzheimer’s disease (65), ischemic stroke, and ICH (9, 66). Based on the regulatory mechanisms of ferroptosis and the pathological processes associated with ICH, targeting iron overload and oxidative stress may prove beneficial in improving ferroptosis of brain tissues (Figure 3). It is well known that the light chain of cystine/glutamate antiporter (system Xc^−^) dysfunction is one of the key pathways in the inducing of ferroptosis, which results in ferroptosis by promoting the accumulation of intracellular lipid ROS. Conversely, the restoration of system Xc^−^ activity has been verified to improve the antioxidant capacity of neurons and inhibit neuronal ferroptosis (67). As reported previous, iron metabolism-related lipocalin-2 (LCN2) has been identified as a mediator of ferroptosis in microglia and nerve injury, acting through the induction of ferritin light chain (FTL) deficiency (68). Moreover, the system Xc^−^ can be inhibited by ICH activated LCN2. Transcriptional and network pharmacological analyses, as well as proteomics analyses, have confirmed that dihydromyricetin can attenuate ferroptosis through reversing the inhibition effect of LCN2 on system Xc^−^ and increasing the GPX4 protein levels (69). The system Xc^−^ and its downstream GSH/GPX4 together constitute a reduction system that deal with the ferroptosis process. The mechanism and application of GPX4 in the ferroptosis process have also attracted considerable attention. It has been demonstrated that exosomes derived from the plasma of healthy young people carrying miR-25-3p can activate the SLC7A11/GPX4 signaling pathway by specifically inducing P53 down-regulation, thereby improving ferroptosis and promoting neurological recovery after ICH (70). Researches of miRNA microarray analysis has confirmed that exosomes derived from mesenchymal stem cells containing miR-25-3p also plays an important role in alleviating neuronal ferroptosis (71). The H63D mutation of the iron homeostasis regulator gene can effectively improve neurological recovery after ICH by activating Nrf2, GPX4 and FTH1 with inducing an antioxidant response (72). In addition, a variety of pharmacological agents, including Netrin-1, Rhein, Angong Niuhuang Wan and others, have been shown to inhibit ferroptosis post-ICH by activating GPX4-related pathways (66, 73, 74).

For abnormal iron homeostasis, another key cause of ferroptosis after ICH, various free iron chelating agents have been highly expected, including deferoxamine and minocycline. In a recent phase 2 clinical trial of deferoxamine for the treatment of ICH, in which 291 patients were enrolled in the analysis of functional recovery assessment for up to 6 months. The results demonstrated that deferoxamine treatment could significantly improve mRS Scores at various time points and the recovery trajectory after ICH (75). This is closely related to the iron chelation properties of deferoxamine and its multiple neuroprotective effects, including antioxidant and anti-inflammatory effects. Furthermore, recombinant lactoferrin has been shown to reduce neuronal ferroptosis in ICH mice by binding to ferric ions (56). In addition to iron overload in the microenvironment of brain tissues in peri-hematoma, the uptake, storage, and utilization of free iron by cells also determine the fate of ferroptosis in brain tissues. In the post-ICH mouse intervention study, apotransferrin intervention did not result in a change to the volume of hematoma or the expression level of GPX4/system Xc^−^. However, it did lead to a reduction in the content of lipid peroxidation secondary metabolites and ferroptosis marker 4-HNE. These results suggest that apotransferrin treatment may provide prognostic benefits in ICH probably related to iron overload and iron metabolic pathways, but not the GSH/GPX4 redox system (76). Consequently, based on the mechanism of ferroptosis following ICH, therapeutic strategies such as the inhibition of free iron accumulation and the activation of the antioxidant defence of the system Xc^−^/GSH/GPX4 axis to reduce ferroptosis may offer novel insights into clinical treatment after ICH. Further improvements in clinical outcomes are anticipated with the development of new therapeutic strategies targeting ferroptosis after ICH.

## 7 Conclusion

Intracerebral hemorrhage is a neurological disease with a high mortality and morbidity rate. The injury mechanism can be divided into two stages: primary mass effect and various secondary brain injury. Secondary brain injury includes a range of processes and substances, such as the formation of hematoma and its degradation products, oxidative stress, inflammatory response, cerebral edema, the coagulation cascade reaction, disruption of the blood–brain barrier, and cell death. The cell death following ICH can occur via apoptosis, necrosis, ferroptosis, or autophagy. Ferroptosis is a form of regulated cell death that was first proposed in 2012 and is an iron and lipid peroxidation dependent cell death form. The brain tissues with a high lipid content will further form an iron overloaded pathological microenvironment after ICH, this has brought the mechanism and intervention research of ferroptosis into focus. Ferroptosis induced cell death after ICH is one of the core mechanisms of secondary brain injury, which forms a vicious cycle through iron overload, lipid peroxidation and the breakdown of antioxidant system, aggravating neuronal death and neurological dysfunction. Intervention strategies targeting ferroptosis, such as iron chelating agents, antioxidants, and gene regulation, have shown significant therapeutic potential in preclinical studies, providing new research directions for improving the prognosis of ICH.

Ferroptosis intervention is of great significance for improving neurological function damage. This article presents an overview of the mechanisms of ferroptosis, as well as the associated injury mechanisms and intervention measures for ferroptosis in the context of ICH. The occurrence of ferroptosis following ICH is closely associated with the hematoma and its subsequent degradation products, oxidative stress, inflammatory response, iron metabolism, and mass effect. Therefore, a range of strategies can be employed to inhibit ferroptosis following ICH. It is hoped that this will provide a comprehensive theoretical basis for the intervention research of ICH. However, future studies should further explore the interaction between ferroptosis and other cell death pathways (e.g., necrosis and apoptosis), and use multi-omics techniques (e.g., single-cell sequencing and metabolomics) to reveal the spatio-temporal dynamic changes of ferroptosis and its cell-specific regulatory mechanisms to optimize precise intervention strategies. Moreover, the effective and rational utilization of various intervention measures is required to be further verified and improved in the process of drug delivery strategy research and clinical practice.
